# Immune cells and RBCs derived from human induced pluripotent stem cells: method, progress, prospective challenges

**DOI:** 10.3389/fcell.2023.1327466

**Published:** 2024-01-05

**Authors:** Jin-he Jiang, Ru-tong Ren, Yan-jie Cheng, Xin-xin Li, Gui-rong Zhang

**Affiliations:** ^1^ Shandong Yinfeng Academy of Life Science, Jinan, Shandong, China; ^2^ Institute of Biomedical and Health Science, School of Life and Health Science, Anhui Science and Technology University, Chuzhou, Anhui, China

**Keywords:** iPSC (induced pluripotent stem cell), iPSC derivation, red blood cell, iPSC derived T cell, iPSC derived NK cell, iPSC derived macrophages cell, iPSC dereved iNKT cell

## Abstract

Blood has an important role in the healthcare system, particularly in blood transfusions and immunotherapy. However, the occurrence of outbreaks of infectious diseases worldwide and seasonal fluctuations, blood shortages are becoming a major challenge. Moreover, the narrow specificity of immune cells hinders the widespread application of immune cell therapy. To address this issue, researchers are actively developing strategies for differentiating induced pluripotent stem cells (iPSCs) into blood cells *in vitro*. The establishment of iPSCs from terminally differentiated cells such as fibroblasts and blood cells is a straightforward process. However, there is need for further refinement of the protocols for differentiating iPSCs into immune cells and red blood cells to ensure their clinical applicability. This review aims to provide a comprehensive overview of the strategies and challenges facing the generation of iPSC-derived immune cells and red blood cells.

## 1 Introduction

In the past century, the demand for blood the social healthcare system has increased tremendously. Despite the existence of numerous blood banks worldwide and over 100 million units of donated blood annually, the currently available blood does not meet global demand for this vital resource. Blood is an essential product in many medical procedures, ranging from surgical interventions for treating chronic diseases such as thalassemia and other forms of anemia. Three main factors contribute to the insufficiencies in blood supplies. Firstly, there is an immense demand for blood. Despite the organization of numerous global blood donation events each year, several factors such as natural disasters, wars and unpredictable outbreaks of infectious diseases increase the blood usage while decreasing opportunity for replenishment. For example, in 2019, the global COVID-19 exacerbated global blood shortage due to increased demand for blood transfusions. Singapore’s blood supplies are down by a third, while 39% of blood centers in the US are only one to 2 days’ supply short (https://www.redcross.org/about-us/news-and-events/press-release/2020/american-red-cross-faces-severe-blood-shortage-as-coronavirus-outbreak-threatens-availability-of-nations-supply.html). The second factor relates to the storage limitations for the donated blood. Anticoagulants are required for proper red blood cells (RBCs) preservation and an appropriate preservation temperature of 4°C ± 2°C is needed (Hess, 2006). Long-term storage causes alteration to the RBC metabolism, reduces erythrocyte viability, degradation of the erythrocyte membrane and increases oxidative stress levels in the stored blood (Orlov and Karkouti, 2015). Transfusion of older donor blood reserves elevates non-transferrin-bound iron (NTBI), which is positively correlated with increased risk of infection and oxidative damage (Kalhan et al., 2017). Therefore, the U.S. Food and Drug Administration (FDA) mandates that RBCs be stored for no longer than 42 days (D'Alessandro et al., 2010). The last challenge lies in the scarcity of potential blood substitutes. Although perfluorocarbon (PFC) emulsions and acellular hemoglobin-based oxygen carriers (HBOCs) have emerged as two key alternatives, their short half-lives and potential adverse effects on physiological systems limit their clinical application, and this warrants further exploration and refinement (Cabrales and Intaglietta, 2013; Modery-Pawlowski et al., 2013).

Cellular therapy has become a major medical biotechnological invention. It is based on groundbreaking scientific discoveries and technological advancements. Although several cellular therapies are still in the experimental stage, treatments such as hematopoietic stem cell transplantation for blood-related disorders (Becker et al., 1963; Weissman and Shizuru, 2008) and anti-CD19 chimeric antigen receptors-T (CAR-T) cell therapy for B-cell malignancies are not widely applied in clinical practice (Neelapu et al., 2017; Schuster et al., 2017; Maude et al., 2018). In immunotherapy, immune cells are isolated from the patient’s peripheral blood and then edited to express chimeric antigen receptors (CARs). These engineered immune cells go through several amplification cycles *in vitro* and subsequently infused back into the patient (Sterner and Sterner, 2021; Jogalekar et al., 2022; To et al., 2022). Notably, natural killer (NK) cells and macrophages exhibit own unique advantages in the treatment of tumors compared to T cells. Based on the success recorded for CAR-T therapy, researchers are now developing CAR-NK cells, CAR-macrophages (CAR-M), and other forms of therapies. Immunotherapy has shown positive effects in the treatment of blood cancers. However, it has certain limitations. Generally, the number of immune cells in the patient’s peripheral blood is in the range of 5%–15%, which may not be sufficient to effectively inhibit tumor cells. Notably, autologous CAR-T cell therapy is costly and its administration is time-consuming. To address these challenges, researchers are attempting to develop off-the-shelf allogeneic CAR-T cells, CAR-NK cells, and NK cells, which may be more readily available for patients (Heipertz et al., 2021; Sadeqi Nezhad et al., 2021; Lu et al., 2022).

Using pluripotent stem cells (PSC) such as induced pluripotent stem cells (iPSCs) and human embryonic stem cells (hESCs) have shown great promise in addressing the need for blood supply and overcoming limitations in immunotherapy. iPSCs have ethical advantages and a more accessible cell source compared to hESCs. Theoretically, iPSCs can be induced to differentiate into any of the three germ layer cells, including immune cells and red blood cells. Today, it is relatively simple to generate iPSCs from various terminally differentiated cells like fibroblasts, blood cells, and somatic cells. Several differentiation protocols have been established for inducing iPSCs differentiation into specific cell types, including immune cells and red blood cells. However, there is need for further refinement of the protocols to improve the clinical utility of such methods. This review provides a comprehensive summary and discussion of strategies for generating immune cells and red blood cells from iPSCs, thereby lay the foundation for researchers working with induced pluripotent stem cells.

## 2 iPSC overview

hESCs were first discovered by James Thomson’s group in 1998, several years after the generation of mouse ESCs (Thomson et al., 1998). These pluripotent stem cells can proliferate indefinitely and differentiate into cells from all three germ layers, making them ideal for developing clinical cell-based therapies.

However, the use of hESCs faces several limitations due to ethical concerns surrounding the utilization of human embryos and the increased risk of immune rejection after transplantation. These factors limits their original intended use in disease modeling and clinical settings. The generation of a single hESC line raised ethical, scientific, and legal considerations, leading to the formulation of laws and policies restricting hESCs use and research funding, particularly in the United States. To address these difficulties, scientists mainly employed two approaches. The first involves somatic cell nuclear transfer (SCNT) (Thomson et al., 1998), which entails using a patient’s own somatic cells for generating hESCs through nuclear transfer (Tachibana et al., 2013). However, there are lingering doubts and apprehensions regarding the outcomes of this method, and the persistently low efficiency of SCNT remains an unresolved challenge. The second strategy involves the derivation of iPSCs, which have become the most commonly applied type of human pluripotent stem cells. In 2006, Nobel Laureate Dr. Shinya Yamanaka and others successfully reprogrammed somatic cells into pluripotent stem cells in mice by introducing four factors: OCT3/4, SOX2, c-MYC, and KLF4 (Takahashi and Yamanaka, 2006). Within a year, human iPSCs (hiPSCs) were successfully generated (Takahashi et al., 2007; Yu et al., 2007). Studies have shown that iPSCs and hESCs are molecularly and functionally similar, displaying no discernible differences at the genetic, epigenetic, or transcriptional levels (J. Choi et al., 2015). Following the establishment of iPSCs, numerous methods have been created to improve the generation efficiency and safety. Some of such methods have also led to the production of footprint-free iPSC lines, which do not require integration of viral vector sequences into their genomes, thereby alleviating concerns regarding genetic modifications during the reprogramming process (Malik and Rao, 2013).

## 3 iPSC-derived immune cells

In the field of cancer immunotherapy, a promising approach called adoptive cell therapy is employed, which involves the injection of potent immune cells into the tumor microenvironment to stimulate anti-tumor responses. Autologous T cells, especially those with modified T cell receptors (TCR) or CAR-T, have elicited positive responses in numerous hematologic malignancies. Despite its potential in treating solid tumors, CAR T cell therapy has limitations that need to be addressed, such as severe life-threatening toxicities, limited tumor infiltration and antigen escape (Sterner and Sterner, 2021). To optimize its efficacy, researchers are exploring alternative cell therapies, including CAR-NK and CAR-M. Compared to CAR T cells, CAR-NK cells offer advantages such as not requiring HLA compatibility and exhibiting lower toxicity when killing cancer cells. Meanwhile, macrophages can efficiently infiltrate tumors, phagocytose target cells, and present tumor antigens, making CAR-M a promising treatment option (Pan et al., 2022; Maalej et al., 2023). In the Table 1, we have listed several immune cell therapy drugs and summarized them based on their respective cell types, targeted diseases, prices and other factors.

**TABLE 1:** Summary of several immune cell therapy drugs.

Name	Cell types	Disease	Autologous or not	Status	Price	References
Tisagenlecleucel (Kymriah)	CAR-T cells	r/r follicular lymphoma (FL) and relapsed or refractory (r/r) diffuse large B-cell lymphoma (DLBCL)	Yes	FDA Approval	475,000 dollars	https://www.drugs.com/kymriah.html
Axicabtagene ciloleucel (Yescarta)	CAR-T cells	large B-cell lymphoma	Yes	FDA Approval	373,000 dollars	https://www.drugs.com/yescarta.html
Brexucabtagene autoleucel (Tecartus)	CAR-T cells	mantle cell lymphoma and acute lymphoblastic leukemia	Yes	FDA Approval	373,000 dollars	https://www.drugs.com/tecartus.html
lisocabtagene maraleucel (Breyanzi)	CAR-T cells	large B-cell lymphoma	Yes	FDA Approval	410,3000 dollars	https://www.drugs.com/breyanzi.html
ciltacabtagene autoleucel (Carvykti)	CAR-T cells	relapsed or refractory multiple myeloma	Yes	FDA Approval	465,000 dollars	https://www.drugs.com/carvykti.html
idecabtagene vicleucel (Abecma)	CAR-T cells	multiple myeloma	Yes	FDA Approval	419,5000 dollars	https://www.drugs.com/abecma.html
ROBO1 CAR-NK cells	CAR-NK cells	Solid tumor	Yes	Clinical trial phase I/II		Maalej et al. (2023)
MUC1 CAR-NK cells	CAR-NK cells	MUC1 positive relapsed or refractory solid tumor	Yes	Clinical trial phase I/II		Maalej et al. (2023)
HER2 CAR- macrophages	CAR-M	HER2 overexpressing solid tumors	Yes	Clinical trial phase I		Maalej et al. (2023)
Glypican 3 (GPC3) CAR-macrophages	CAR-M	Solid tumors	Yes	Clinical trial phase I		Maalej et al. (2023)

However, some factors hinder the widespread clinical application of current autologous therapies. These include high costs, difficulties in large-scale manufacturing, and limited accessibility for patients with low lymphocyte counts. To overcome these barriers, iPSCs are considered an ideal source of cells for engineering off-the-shelf allogeneic cell therapies.

The main advantages of iPSCs include their unlimited capacity for expansion, relative ease of genetic engineering, the ability to select specific clones after genetic modification, and the elimination of the need to collect cells from donors at any particular time. Consequently, iPSCs are considered a suitable source of cells for generating allogeneic cell therapies for use. These iPSCs can be differentiated into immune cells which can be employed to develop therapies for cancer treatment.

### 3.1 iPSC-derived T cells

Cytotoxic T lymphocytes (CTLs) can recognize and eliminate infected cells as well as malignant host cells. CAR-T cell therapy utilizes the body’s immune system to achieve targeted treatment against tumors (Sterner and Sterner, 2021). Currently, over 1,000 clinical trials are being conducted on CAR-T cell therapy. Despite notable clinical efficacy has been reported so far, some limitations have been noted, including its high cost, risk of preparation failure, and protracted pre-treatment lead times (Halim and Maher, 2020; Fischer and Bhattarai, 2021; Sanber et al., 2021). Consequently, allogeneic CAR-T cells should be developed as a viable solution. Such an approach would streamline the manufacturing process, standardize production, and increase their clinical application (Caldwell et al., 2020; Depil et al., 2020). Moreover, this would obviate the need for individualized cell preparation and promote advanced production of CAR-T cells, making them accessible to multiple patients and allow repeated treatments whenever necessary.

#### 3.1.1 The method and progress of iPSC-derived T cells

**FIGURE 1:**
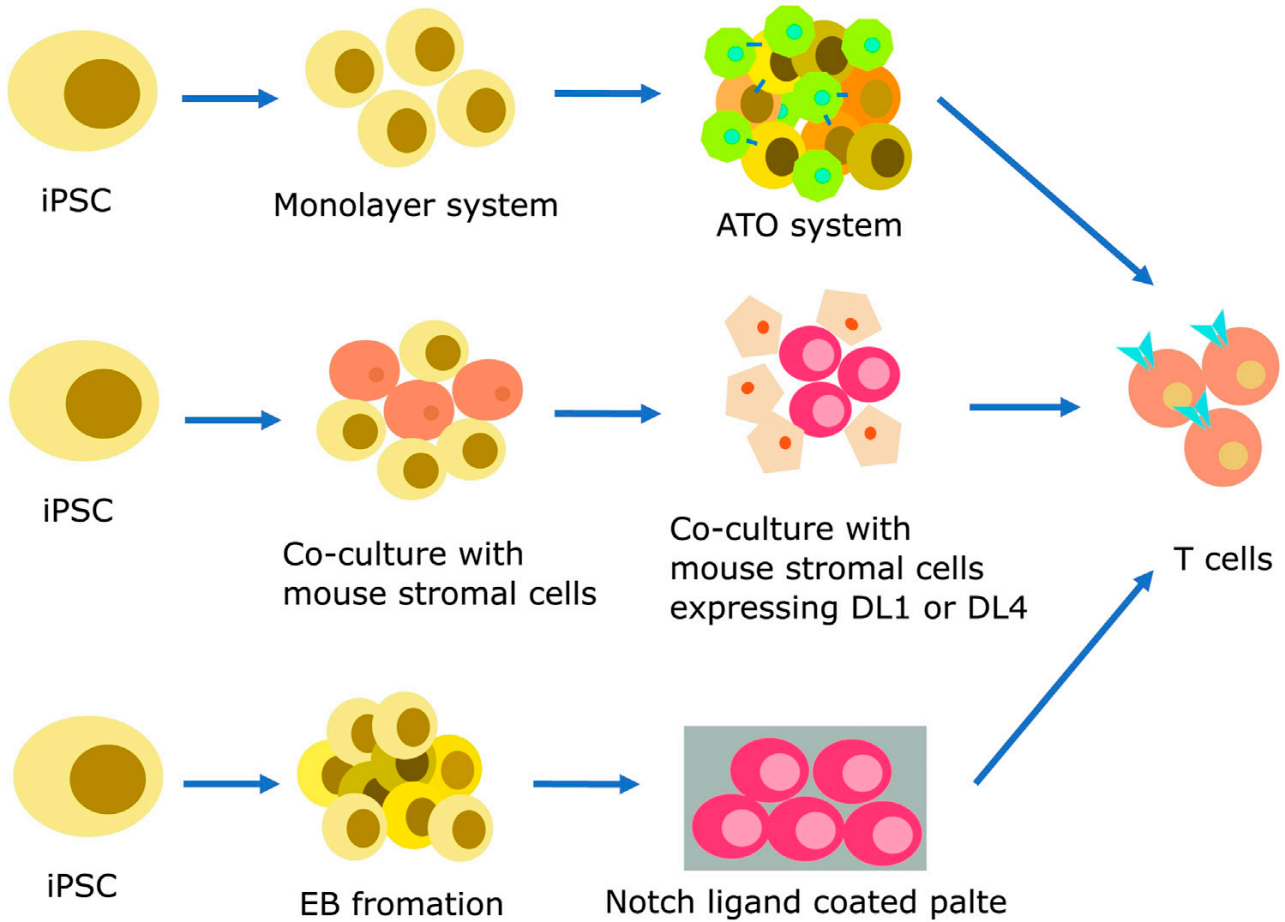
Strategies for producing iPSC-derived T cells. Hematopoietic differentiation can be initiated using various protocols, including feeder-free methods like monolayer systems, co-culture with mouse stromal cells, and EB formation. At this stage, endothelial-to-hematopoietic transition, CD34^+^ HSPCs emerge from the HE layers. The specification of T cell lineage requires Notch signaling, which can be made easier by co-culture with mouse stromal cells such as OP9-DL1 or OP9-DL4. Mature T cells can be efficiently generated by co-culturing iPSC-derived multipotent HSPCs with these cells in a 2D or 3D system. A coating matrix mixture that contains retronectin and recombinant DL4 protein can be used to provide Notch signals as an alternative.

Inducing iPSCs into T cells presents a feasible approach. The standard procedure for producing iPSC-derived T cells encompasses a multi-step process, which includes mesoderm specification, hematopoiesis initiation, and T cell differentiation (Themeli et al., 2013; Kawamoto et al., 2021; Netsrithong and Wattanapanitch, 2021; Xue et al., 2023). Initially, PSCs must be directed towards HSCs. In this phase, iPSCs undergo an endothelial-to-hematopoietic transition (EHT) process, which encompasses the generation of definitive mesoderm (ME) and hemogenic endothelium (HE). iPSCs can be differentiated into HSCs using three distinct techniques: embryoid body (EB) formation (Kennedy et al., 2012; Themeli et al., 2013), feeder-free procedures like the monolayer system (Netsrithong et al., 2020; Suwanpitak et al., 2022), and co-culture with mouse stromal cells (Timmermans et al., 2009). After successful HSC induction, iPSCs are then guided toward the T cell lineage commitment through the activation of Notch signaling pathway (Besseyrias et al., 2007). The differentiation of T cells can be achieved using three different strategies as Figure 1.

The co-culture method which involves the use of stromal cells is a simple and well-known approach for inducing T cell differentiation from iPSCs. In this method, iPSCs derived from healthy body cells are co-cultured with murine bone marrow stromal cells such as C3H10T1/2 or OP9 cells. This co-culture results in the formation of CD34^+^ hematopoietic progenitor cells (HPCs). The CD34^+^ HPCs are then co-cultured with OP9 cells overexpressing delta-like 1 (DL1) or delta-like 4 (DL4), along with cytokines such as IL-7 and IL-3, inducing their differentiation into T cells (Karanu et al., 2001; Schmitt and Zuniga-Pflucker, 2002; La Motte-Mohs et al., 2005). Notably, the T cell yield remained relatively consistent when co-cultured with OP9 cells expressing DL1 or DL4. However, compared to DL1, DL4 demonstrated a tenfold increase in Notch receptor binding (Mohtashami et al., 2010; Andrawes et al., 2013). Subsequent stages encompass the differentiation of HSC cells co-cultured with OP9-DL1 cells in the presence of Flt3L and IL-7, leading to the production of human pro-T cells. These pro-T cells can be activated either using anti-CD3 antibodies or effector T cells from peripheral blood, ultimately resulting in the generation of mature T cells (Nishimura et al., 2013; Vizcardo et al., 2013; Maeda et al., 2016).

The artificial thymic organoid (ATO) is an innovative approach for inducing iPSCs differentiation into T cells. The ATO system simulates the thymus environment using mouse MS5 cells expressing the human DL1 or DL4 in a serum-free medium. In previous studies, TCR αβ and CD3 positive T cells were generated without the use of anti-CD3 antibodies through the ATO system as reported by Seet et al. (Seet et al., 2017). In a recent study by Wang et al., in 2022, iPSCs reprogrammed from CD62L naive and memory T cells were induced to differentiate into T cells using a 3D organoid system (Wang et al., 2022). T-iPSC clones were transduced with lentivirus encoding a CD19-targeting CAR. After sorting, the cells were cultured in the absence of feeder cells. CD56^+^CD326-iPSC mesodermal progenitor cells were subjected to co-cultivation with the MS5 cell line overexpressing hDL4 to initiate differentiation into HPCs. The process of T cell differentiation occurred within the ATO system, utilizing a serum-free medium supplemented with factors like SCF, Flt-3L, hIL-7, B27, among others. Finally, iPSC-derived T cells were expanded using irradiated PBMC and LCL cells in X-VIVO15 medium supplemented with the anti-CD3.

To resolve the limitations associated with using mouse cell lines as feeder cells in generating iPSC-derived T cells, several feeder-free differentiation systems have been developed. The use of mouse feeder cells increases the risk of cross-species contamination and makes it difficult to perform quality control. To overcome this, Iriguchi et al. established a feeder-free differentiation culture system in 2021 which covers the entire process from iPSC maintenance to T-cell proliferation (Iriguchi et al., 2021). Specifically, they incorporated immobilized DL4 protein and retronectin to replace feeder cells. This system enabled large-scale production of regenerated T cells. Elsewhere, a study by Ito et al. employed a feeder-free protocol to establish tumor-specific TIL-derived iPSCs using human colorectal cancer specimens [54]. T cells differentiated from TIL-iPSCs, called TIL-iPS-T, retained intrinsic T cell functions and tumor specificity (Ito et al., 2021). However, the aforementioned methods still involved the use of bovine serum albumin (BSA). BSA is commonly used in cell culture media as a protein source and can support cell growth and differentiation.

#### 3.1.2 The challenges of iPSC-derived T cells

Despite the publication of many protocols, challenges persist in the field of generating differentiated T cells with adequate quality control. To ensure product safety, it is important that the differentiation protocol for producing T cells be developed using a xenogeneic-free system. Although some researchers had made the improvement, one major challenge is still that most existing protocols involve the use of serum and murine feeder cells.

Adoptive T cell immunotherapy relies on the unique tumor antigen recognition capabilities of T cells, which is naturally conferred by the expression of certain TCRs encoded by specially rearranged genomic loci of the TCRα and β chains. However, when T cells are produced from iPSCs, random rearrangements of TCRα and β chains can result in a population of cells with unknown specificities, as differentiated T cells bear non-rearranged germline TCR loci (Timmermans et al., 2009). Cytotoxic CD8 single-positive (SP) T cells, generated from iPSCs derived from peripheral blood T lymphocytes, are able to identify target antigens on cell lines and exhibit potent cytotoxicity (Themeli et al., 2013; Maeda et al., 2016). The final and critical step in the protocol involves generating mature CD8 αβ+ cells. The protocol of T cells induced from iPSCs is a complex and multi-step process with varying efficiency. Another challenge is the production of mature SP T cells suitable for clinical applications.

### 3.2 iPSC-derived NK cells

NK cells were the first type of innate lymphoid cells to be discovered (Kiessling et al., 1975a; Kiessling et al., 1975b). NK cells have distinct advantages compared to T cells in the context of adoptive cell therapy. Although T cells possess potent specific cytotoxicity and high expansion capacity, NK cells have distinct characteristics. NK cells are capable of directly eliminating tumor cells through processes like releasing cytotoxic granules and triggering apoptosis. However, they may exert milder effects compared to T cells, which can be advantageous in specific situations (Lee, 2019). One key advantage of NK cells is that they undergo a process of re-education in the host body. This phenomenon minimizes the risk of graft versus host disease (GVHD). Therefore, allogeneic (donor-derived) NK cells can be used in adoptive cell therapy without eliciting strong immune reactions against the recipient’s tissues (Ruggeri et al., 2002). On the basis of these properties, allogeneic NK cells have attracted significant attention in the field of adoptive cell therapy and are commonly applied in many spheres. Their ability to target tumor cells and reduce the risk of GVHD makes them an attractive option for developing immunotherapies.

In the early stages of cell therapy, NK cells were primarily obtained from peripheral blood (PB). However, alternative sources of NK cells which are more advantageous in terms of abundance and ease of modification have been identified. The umbilical cord blood (CB) is a rich source of NK cells and has been found to have a higher blood supply compared to PB (Shankar et al., 2020; Myers and Miller, 2021). Additionally, NK-92 cell lines, which are derived from a patient with non-Hodgkin’s lymphoma, serve as a continuous cell line that is easy to expand and modify in therapeutic applications (Shankar et al., 2020; Myers and Miller, 2021). Another promising approach involves the use of stem cells, specifically iPSCs, for generating NK cells. iPSCs have several advantages in this context. They can be derived from various cell types and can differentiate into any cell type, including NK cells. Therefore, they provide an ideal platform for developing allogeneic NK cell-based therapies. By creating stable engineered iPSC lines, researchers can generate a uniform population of iPSC-derived NK cells that possess the desired characteristics in therapeutic applications. Techniques have been devised to cultivate, genetically modify, and guide iPSCs toward becoming functional NK cells. This allows for the generation of abundant, standardized, and precisely characterized cell populations for therapeutic purposes. The utilization of iPSC-derived NK cells presents significant potential in augmenting the efficacy and versatility of adoptive cell therapy, opening up novel avenues for addressing a range of diseases, including cancer.

#### 3.2.1 The method and progress of iPSC-derived NK cells

**FIGURE 2:**
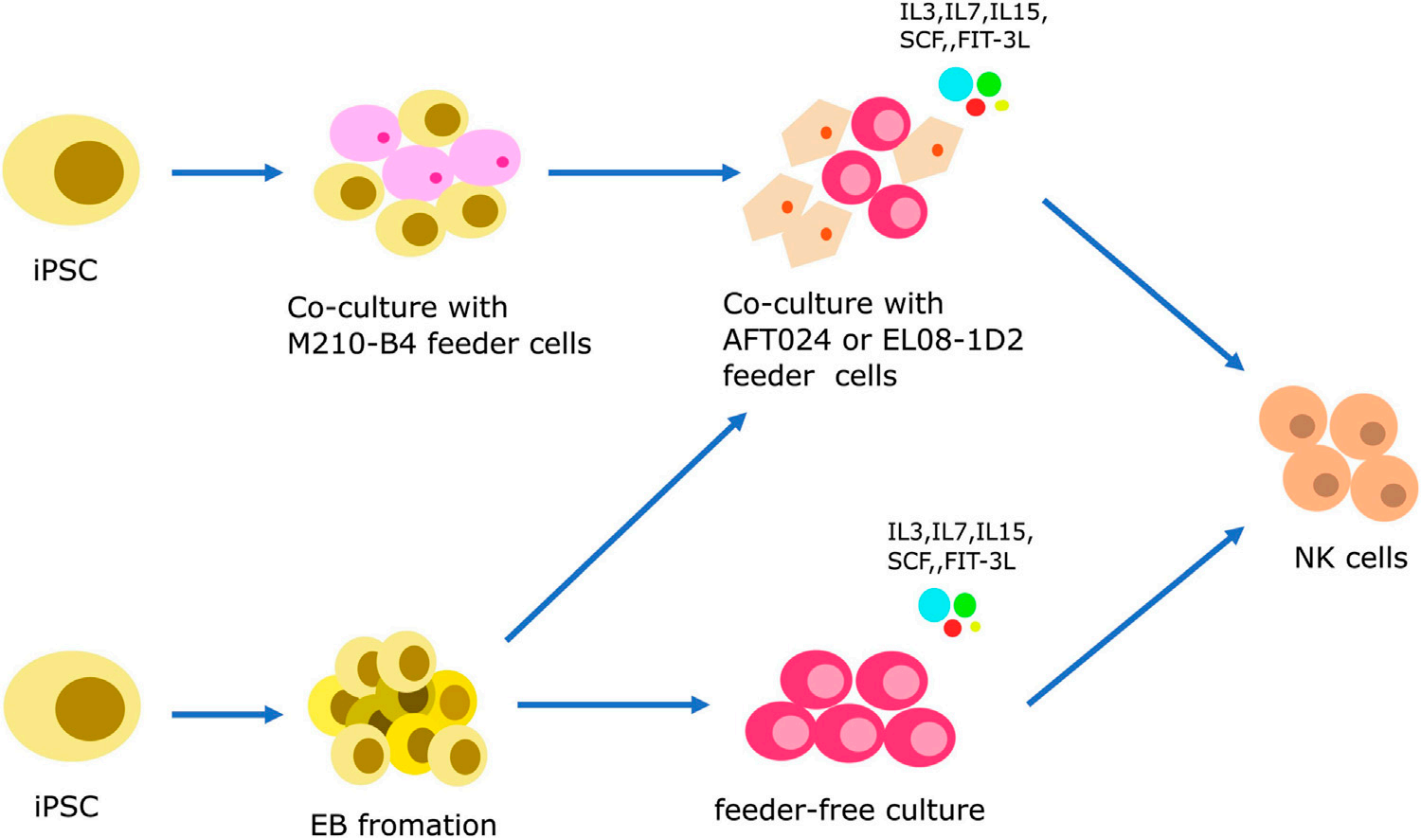
Strategies to generate iPSC-derived NK cells. iPSCs are induced to initiate hematopoietic differentiation through co-culturing with mouse feeder cells (M210-B4 cells) or by forming embryoid bodies (EB). During this stage, CD34^+^ HSPCs emerge from the HE layers, similar to the protocol for iPSC-derived T cells. The specification of NK cell lineage is then achieved either through a feeder-dependent system or a feeder-independent system.

Similar to iPSC-derived T cells, iPSC-derived NK cells are being investigated as a means of generating homogeneous populations for off-the-shelf allogeneic therapies. The differentiation protocols for PSC-NK cells typically involve two systems: a 2D culture system and a 3D culture system (Figure 2). Both include two stages, i.e., the induction of hematopoietic differentiation and HPCs differentiation into NK cells.

Prior to the discovery of iPSCs, hESCs have been the primary source of stem cells. The protocols used to generate iPSC-derived natural NK cells have been adapted from the differentiation protocols used for hESCs (Romee et al., 2016; Hsu et al., 2021). In 2011, the Kaufman group described a two-step feeder-dependent culture approach for generating iPSC-NK cells. The initial stage entailed co-cultivating iPSCs with the mouse stromal cell line M210-B4 for a duration of 19–21 days. This co-cultivation was designed to facilitate the development of hematopoietic stem cells (HSCs) from the iPSCs (Ni et al., 2011). In the subsequent step, CD34^+^ hematopoietic progenitors, derived from the iPSCs, were isolated and subsequently co-cultivated with a murine stromal line known as AFT024. This co-culture was enriched with a cocktail of cytokines comprising of SCF, Flt-3L, IL-3, IL-7, and IL-15, and lasted for 4-5 weeks. This stage was designed to stimulate the maturation of the HSCs into functional NK cells (Ni et al., 2011). The method described by the Kaufman group successfully established functional NK cells from both hESCs and iPSCs. However, the use of murine-derived feeder cells, such as M210-B4 and AFT024, limits the clinical application of this method. To overcome this limitation, it is imperative to explore alternative approaches for eliminating the need for feeder cells to generate iPSC-NK cells for clinical use.

In 2013, the Kaufman research group established a feeder-free 3D culture method using EB formation with the aim of overcoming the limitations associated with feeder cells. When iPSCs are cultured in suspension without feeder cells, they form aggregate structures called embryoid bodies. The formation of EB creates the challenge of size variations, which affect the differentiation and compliance with Good Manufacturing Practice (GMP) standards (Bock et al., 2013). To address this issue, the Kaufman group introduced spin-EBs, which involved the centrifugation of known numbers of iPSC-EBs to achieve uniform size. In this procedure, iPSCs are cultured without feeder cells for a week, counted, and seeded at a concentration of 3,000 cells per well in a round-bottom 96-well plate to facilitate EB formation. Feeder-free media supplemented with BMP4 and SCF is used to induce HPCs differentiation in the EBs. At day 11 of the spin-EB differentiation process, six wells from the 96-well plate are transferred directly to one well of a 24-well plate. These cells are then cultured with NK cell-initiating cytokines, including SCF, Flt-3L, IL-3, IL-7, and IL-15, to promote the formation of NK cells. The resulting iPSC-derived NK cells can be further expanded through several NK cell culture cycles. Notably, the phenotypes and activity of these cells are comparable to those of natural NK cells (Woll et al., 2009; Goldenson et al., 2020). In 2019, the Kaufman group further modified this method by introducing a strategy that eliminates the need for single-cell adaptation of hESCs and hiPSCs, which had previously taken months to achieve. Specifically, they used a Rho-associated protein kinase inhibitor (ROCKi) to directly induce the formation of EB (Zhu and Kaufman, 2019).

#### 3.2.2 The challenges of iPSC-derived NK cells

Although iPSC-derived NK cell protocols have significantly improved the field of cell therapy, they have some limitations, particularly when considering their application in clinical settings. One of the challenges lies in the complexity and variety of cytokines and compounds involved in the differentiation process of iPSCs into NK cells. The precise administration of these factors in terms of dosage and timing is of utmost importance and demands stringent control. Ensuring a consistent and reproducible process for manufacturing iPSC-derived NK cells presents a significant challenge. In conventional methods, there exists a time-intensive single-cell adaptation phase that can extend over several months. Although the 2019 protocol developed by Kaufman group could bypass this step, the overall time required for generating reliable adoptive cell therapies using iPSC-derived NK cells is still lengthy (Zhu and Kaufman, 2019). This time factor hinders the translation of iPSC-derived NK cells into effective treatments for patients. To surmount these hurdles, future progress may unfold as biopharmaceutical companies refine the protocols for producing iPSC-derived NK cells on a clinical scale. These developments have the potential to streamline and enhance the process, rendering it more viable for widespread application in adoptive cell therapy.

### 3.3 iPSC-derived macrophages cells

Macrophages are an important component of the innate immune system. They are distributed in different tissues and organs during development and their expression is maintained throughout life through local proliferation and homeostatic recruitment (Wynn et al., 2013). Functionally, macrophages respond to microenvironmental signals in the specific tissues, which then polarize into two primary phenotypes: “classically activated” M1 macrophages and “alternatively activated” M2 macrophages (Donadon et al., 2020). M1 macrophages are induced by Th1 interferon (IFN-γ and TNF-α) as well as lipopolysaccharides (LPS). They can directly phagocytose and eliminate pathogens in peripheral tissues, together with pro-inflammatory cytokines such as TNF-α, IL-1α, IL-1β, IL-6, IL-12, and IL-2. Moreover, M1 macrophages participate in antigen processing and presentation to T lymphocytes, further enhancing immune responses. On the other hand, “alternatively activated” M2 macrophages are induced by interleukin (IL)-4, IL-13, or IL-10. These macrophages have limited antigen-presenting ability but exhibit potent immunoregulatory functions by secreting suppressive cytokines like IL-10 or TGF-β, thereby suppressing immune responses (Shapouri-Moghaddam et al., 2018). Moreover, M2 macrophages play a substantial role in tissue regeneration and the process of wound healing. Alterations in macrophage functionality or imbalances between their pro-inflammatory and anti-inflammatory functions can lead to various diseases, such as cancer and cardiovascular disorders. This underscores the significance of macrophages as attractive targets for therapy and as potential candidates for cell-based treatments. It is important to highlight that continuous research in the realm of macrophage biology is refining our comprehension and uncovering the broader therapeutic possibilities associated with these cells.

Three primary models are commonly employed to study human macrophages. The first model involves the isolation of macrophages directly from human tissues. However, this approach is limited by the scarcity of human tissue samples available for research purposes. The second model leverages on immortalized cell lines such as THP-1 or U937 cells. These cell lines, which are derived from malignant cells, cannot accurately represent the diverse nature of macrophages in the human body. Moreover, their low biological relevance makes them unsuitable as drug testing models. The final model entails producing macrophages from monocyte-derived macrophages (MDMs). This method holds notable advantages, as monocytes can be readily obtained from peripheral blood samples, ensuring a high degree of biological relevance. Nonetheless, MDMs come with their constraints, as they cannot be sustained in culture for prolonged periods and pose challenges for genetic modification. Despite these challenges, MDMs are considered the primary source of human macrophages for research purposes. In recent years, new methods for generating and refining macrophages from iPSCs have been developed. These methods are designed to overcome the limitations of previous models by utilizing iPSCs, which can be generated from various cell sources and differentiated into macrophages. This approach can also resolve the challenges associated with other models and provides new avenues for studying human macrophages.

#### 3.3.1 The method and progress of iPSC-derived macrophages cells

The differentiation of macrophages derived from iPSC comprises 4 main stages. 1) mesoderm/HE induction, 2) Hematopoietic differentiation, 3) Myeloid specification, 4) terminal differentiation of macrophages derived from iPSC differentiation (Gutbier et al., 2020; Klepikova et al., 2022; Hang et al., 2023). Macrophages are derived from iPSC through four main types of approaches. One of these approaches involves the use of OP9 cell line as a feeder for differentiation. iPSCs are co-cultured with the OP9 cell line to generate hematopoietic progenitors (Lynch et al., 2011). These progenitors are then induced to differentiate into monocyte-like cells and subsequently into macrophages derived from iPSC using M-CSF or GM-CSF (Brault et al., 2014; K. D. Choi et al., 2009; Kambal et al., 2011; Senju et al., 2011). The use of xenogeneic cells in this method increases the risk of cross-species contamination and reduces protocol standardizability. As a result, this approach is not commonly used to generate macrophages from iPSC, especially in clinical applications.

**FIGURE 3:**
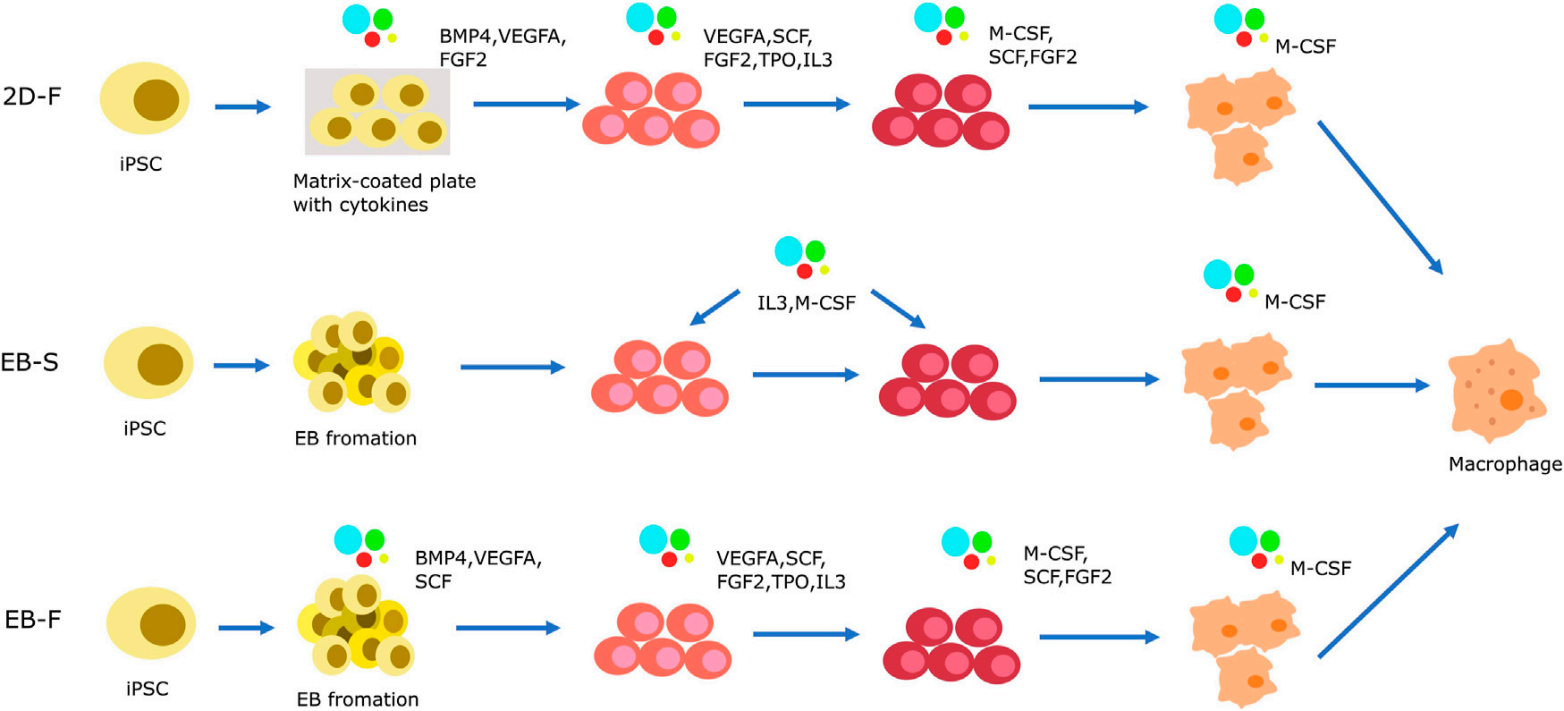
Strategies of iPSC-derived macrophage cells generation in OP9-independent protocol. The differentiation of macrophages derived from iPSC has 4 main stages. (1) mesoderm/HE induction, (2) Hematopoietic differentiation, (3) Myeloid specification, (4) terminal differentiation of macrophages derived from iPSC differentiation. Mesoderm/HE induction are generated by 2D-F protocol or 3D protocol. In 2D-F protocol, iPSCs are cultured in M atrix-coated plates with complex mixes of exogenous factors to driver cells differention. In 3D protocol, the step is completed by EB formation. Based on the presence or absence of exogenous factors, it is divided into EB-S protocol and EB-F protocol. In the 2D-F protocol, a series of exogenous factors with complex compositions is introduced in a sequential manner to orchestrate the progression of cells towards hematopoietic progenitor formation and subsequent myeloid specification. EBs are initially generated and subsequently transferred onto tissue culture plates using the EB-S protocol. These EBs are then cultured with interleukin-3 (IL-3) and macrophage colony-stimulating factor (M-CSF), which synergistically induce the emergence of hematopoietic progenitors and their directed commitment towards the myeloid lineage. Alternatively, the EB-F protocol allows for EB induction either through IL-3 and M-CSF supplementation or through the strategic utilization of a composite mixture comprising various exogenous factors.

In the OP9 cell line-independent protocol (Figure 3), there is no requirement for xenogeneic cells. iPSCs are cultured on matrix-coated plates using a two-dimensional (2D) factor-assisted approach (2D-F). The introduction of various exogenous substances, such as SCF, IL-6, IL-3, and others, aids in the differentiation process. These factors are added in a specific sequence to encourage cell differentiation (Yanagimachi et al., 2013; Takata et al., 2017; Cao et al., 2019; Cui et al., 2021). However, the 2D environment in these protocols constrains the differentiation of iPSCs into the three germ layers, potentially limiting their capacity to completely mimic the intricacy of native macrophages.

Therefore, scientists have developed three-dimensional (3D) protocols using EBs to induce the differentiation of iPSCs into macrophages. Similar to the iPSC-derived NK cells, these protocols involve the formation of mesoderm/HE in the first stage using EBs. The 3D protocol involves two distinct stages, namely EB-S and EB-F, which are based on mesoderm/HE formation. In the EB-S group, iPSCs are cultivated in low-adherent dishes, enabling them to naturally aggregate and form EBs. These EBs can differentiate into all three germ layers, including mesoderm and endoderm. During the mesoderm/HE formation, the addition of cell cytokines is not required. After the mesoderm/HE formation stage, the mesoderm/HE cells are then cultured with selected cell cytokines including IL-3 and M-CSF for hematopoietic differentiation, myeloid specification, and terminal differentiation into induced macrophages (Panicker et al., 2012; van Wilgenburg et al., 2013; Ackermann et al., 2018). In the EB-F group, scientists introduce supplementary mesoderm/HE-inducing factors like BMP4, VEGFA, and SCF to the EBs. These substances play a crucial role in guiding the EB differentiation path and enhancing the efficacy of mesoderm/HE formation. As for the subsequent phases of hematopoietic differentiation and myeloid specification, researchers have pursued various strategies. Some opt to rely solely on IL-3 and M-CSF as exogenous factors for the differentiation of hematopoietic and myeloid cells (Sacks et al., 2018; Gutbier et al., 2020; Lopez-Yrigoyen et al., 2020). This simplified approach facilitates the successful differentiation into the desired cell types. On the other hand, some researchers have utilized a more complex combination of exogenous factors in the hematopoietic differentiation and myeloid specification stages (Zhang et al., 2015; Joshi et al., 2019; Shi et al., 2019). These additional factors include VEGFA, SCF, FGF2, FLT3L, TPO, IL-3, and M-CSF. In the majority of protocols, M-CSF and supplementary cytokines are employed in the terminal differentiation of iPSC-derived macrophages. Through diverse techniques, it is feasible to generate macrophages from iPSCs that display a typical macrophage phenotype and perform essential macrophage functions (van Wilgenburg et al., 2013; Alasoo et al., 2015; Zhang et al., 2015; Ackermann et al., 2018; Haake et al., 2020).

#### 3.3.2 The challenges of iPSC-derived macrophages cells

Each method for generating macrophages from iPSCs has its advantages and considerations. Among such methods, the EB-F group in the EB formation protocol has been found to have good performance in terms of reproducibility, efficiency, and cost-effectiveness. It offers a higher mesoderm/hepatic endoderm formation efficiency and is relatively more cost-effective compared to the EB-S group. The EB-S approach might be considered more economical since it doesn’t necessitate extra mesoderm/HE-inducing factors. Nevertheless, it relies on feeder cells and serum, which can introduce complexity and variability into the culture process. Conversely, the 2D protocol offers benefits like reduced factor consumption, heightened reproducibility, and straightforwardness. However, it results in the formation of fewer macrophages from iPSCs and is more costly. To improve the macrophage differentiation protocols from iPSCs, several issues need to be addressed. This includes increasing cell yield while minimizing costs by adopting optimized strategies. Furthermore, the protocols should be modified to meet clinical requirements. Irrespective of the chosen approach, notable progress has transformed iPSC-derived macrophages into valuable resources for investigating macrophage biology and exploring therapeutic uses. Ongoing refinements in these protocols hold the potential to increase cell yield, lower expenses, and better meet clinical requirements.

### 3.4 iPSC-derived iNKT cells

Invariant natural killer T (iNKT) cells represent a distinct population of innate-like T lymphocytes, often referred to as type I or classical NKT cells. Similar to conventional αβ T cells, they express a conserved TCR chain encoded by the d Vα24 Jα18 gene segment in humans. Moreover, they originate from a common lymphoid progenitor in the thymus (Hogquist and Georgiev, 2020). However, the iNKT cells have a different recognition mechanism. While conventional αβ T cells recognize peptide antigens presented by MHC class I/II complexes, iNKT cells recognize glycolipids presented by CD1d, an MHC-I-like molecule. The activating ligand commonly used for iNKT cells is α-galactosylceramide (α-GalCer). Following activation, iNKT cells secrete several types of cytokines which act as a bridge for the innate and adaptive immune systems. One notable feature of iNKT cells is their ability to target tumor-associated macrophages. They express a higher number of chemokine receptors compared to regular T cells, enabling them to infiltrate tumors and recruit other immune effector cells (Y. Liu et al., 2022). Furthermore, iNKT cells confer protection against GVHD without the limitation of polymorphic human leukocyte antigens (HLAs) (Mavers et al., 2017). These unique innate characteristics and properties make iNKT cells an ideal tool for developing cell-based therapies. However, the concentration of iNKT cells is very low (0.01%–1% of peripheral blood mononuclear cells) in the peripheral blood, which limits their therapeutic applications. To resolve this limitation, the generation of iPSC-derived iNKT cells has been proposed as an effective approach of increasing the yield and availability of these cells for cell-based therapies.

#### 3.4.1 The method and progress of iPSC-derived macrophages cells

The concept of using iPSCs to generate iNKT cells was initially tested in mice and later extended to humans. Watarai et al. pioneered the successful combination of iPSCs and iNKT cells (Watarai et al., 2010). They derived iPSCs from embryonic fibroblasts from an iNKT mouse clone and mouse splenic NKT cells. Using the OP9-DL1 culture system containing cytokines such as IL-15 and IL-7, they successfully differentiated iPSCs into iNKT cells *in vitro*. The iPSC-derived iNKT cells exhibited noteworthy anti-tumor effects through the secretion of the Th1 cytokine IFN-γ, effectively suppressing tumor growth *in vivo*. Building on this successful demonstration in mice, Yamada et al. expanded their research by re-differentiating iPSCs into functional iNKT cells, using human iNKT cells as an intermediate step. This involved culturing PBMCs with α-GalCer and human IL-2 to facilitate iNKT cell expansion (Yamada et al., 2016). The iNKT cells were subsequently reprogrammed to generate iPSCs through infection with a Sendai virus expressing KLF4, OCT4, SOX2, and c-MYC. Each iPSC colony was assessed for its ability to differentiate into three germ layers and gene expression profiles. Using the same culture system, the iPSCs were successfully re-differentiated into functional iNKT cells *in vitro*. The iNKT-derived iPSCs exerted a strong anti-tumor effect and generated similar levels of IFN-γ compared to iNKT cells isolated from PBMCs. They also expanded in response to hIL-7/hIL-15 stimulation but did not produce IL-4. Building upon the groundwork established by preclinical studies, a groundbreaking clinical trial utilizing GMP-grade iPSC-iNKT cells for head and neck tumor treatment was initiated in 2020, under the supervision of Professor Motohashi in Japan (Aoki et al., 2023). The trial is projected to conclude by March 2024 and seeks to furnish compelling evidence on the viability of differentiating human iPSCs into iNKT cells for cellular therapy.

#### 3.4.2 The challenges of iPSC-derived macrophages cells

Compared to the differentiation of iPSCs into T cells and NK cells, the differentiation of iPSCs into iNKT cells has received less attention in research. Similar to the differentiation of iPSCs into other cell types, there are typical limitations in the differentiation of iPSCs into iNKT cells, such as low efficiency, instability, high cost, and complexity. In light of these limitations, current research is continuously striving to enhance the differentiation of iPSCs into iNKT cells to overcome these challenges.

## 4 Red blood cells

There has been a huge shortage of blood supply worldwide due to various reasons (Roberts et al., 2019). Therefore, researchers have attempted to explore *in vitro* blood expansion techniques and search for effective blood substitutes. PFCs and HBOCs are the two commonly studied blood substitutes, but they have demonstrated negative health effects when applied *in vivo*. In the absence of suitable blood substitutes, early studies focused on *in vitro* expansion of hematopoietic stem and progenitor cells (HSPCs) as a potential solution. In 2002, Neildez-Nguyen et al. successfully expanded RBCs from cord blood-derived HSPCs *in vitro*, and achieved an increase of approximately 200,000-fold in pure erythroid populations (Neildez-Nguyen et al., 2002). Subsequently, Baek et al. refined this protocol by utilizing murine MS-5 stromal cells (Baek et al., 2008). In 2006, Nakamura’s research team pioneered a stroma-free approach that resulted in a remarkable 5.8 × 10^^5^-fold expansion of erythroid populations (Miharada et al., 2006). Since then, various research groups have worked to refine these protocols, aiming to further boost the *in vitro* expansion of CD34^+^ HSPC populations. Nevertheless, there are certain limitations associated with these methods. They are highly reliant on donor availability and dedicated blood banks, which can increase batch-to-batch variability. Moreover, ensuring the quality and consistency of CD34^+^ HSPCs remains a challenge that needs to be addressed in further studies.

hPSCs, including hiPSCs, have been considered ideal materials for *in vitro* expansion of RBCs. The use of hiPSCs for RBC creation is expected to increase compared to hESCs due to the moral and political controversies associated with hESC research. The *in vitro* generation of hematopoietic cell types from hiPSCs offers a controlled and uninterrupted manufacturing process for the development of advanced drugs and therapeutics. Selecting hematopoietic stem cells and effector cells derived from hiPSCs with care for transplantation or transfusion purposes can help minimize potential risks related to alloimmunization and the transmission of blood-borne diseases. Furthermore, this approach allows the generation of expanded blood cells expressing blood-enhancing molecules, which may have high potential for clinical applications.

### 4.1 The method and progress of hiPSCs-derived red blood cells

Several protocols have been developed for generating HSCs from hiPSCs based on embryonic development. In 2010, Lapillonne et al. successfully differentiated hiPSCs into erythrocytes using a two-step process: i) differentiation of hiPSCs by forming human EBs, and ii) further differentiation and maturation into mature cultured RBCs in the presence of cytokines (Lapillonne et al., 2010). In this study, it was observed that RBCs derived from hiPSCs showed a restricted capacity for enucleation when compared to hESCs. Following this, the team enhanced the enucleation capability up to 50% by inhibiting miR30A, a microRNA. This led to the production of fully functional and mature RBCs expressing both fetal and adult hemoglobin. Another research groups also showed that hiPSCs can differentiate into erythrocytes *in vitro* using an EB formation protocol (Kobari et al., 2012; Park et al., 2020; Roh et al., 2022).

The EB differentiation protocol provides a 3D microenvironment that is conducive to the enucleation of erythroblasts. However, large-scale production using this protocol can be challenging given its labor-intensive nature. In 2013, Smith et al. established a chemically-defined, serum and feeder cell-free culture system for the production of hiPSCs and their differentiation into megakaryocyte and erythroid lineage cells (Niwa et al., 2011). They identified specific combinations of cytokines such as BMP4, VEGF, WNT3A, FGF2, and aryl hydrocarbon receptor (AhR), which caused the rapid expansion of HPCs, megakaryocyte-lineage cells, and erythroid-lineage cells (Niwa et al., 2011). Other research groups have attempted to optimize this protocol using different factors and small molecules (Niwa et al., 2011; Chou et al., 2012). In 2020, Tursky et al. compared various protocols for generating hematopoietic cells from hiPSCs in terms of efficiency, cost, and time (Tursky et al., 2020). They concluded that the 2D-multistep protocol developed by Smith et al. was the most efficient (Tursky et al., 2020). Other research groups have reported various approaches for improving the EB differentiation protocol to increase production. Olivier et al. modified the EB differentiation process by utilizing a serum and feeder-free approach, achieving an erythroid expansion of 5 × 10^4 to 2 × 10^5 fold (Olivier et al., 2016). Elsewhere, Sivalingam et al., in 2018 optimized the upstream process by modulating the Wnt/β-Catenin signaling pathway. They achieved a substantial expansion of erythroid cells, increasing their numbers up to 10,000-fold. Additionally, they attained an enucleation rate ranging from 28% to 40.6% by employing co-culturing techniques with human mesenchymal stem cells (hMSCs) (Sivalingam et al., 2018). In 2020, Sivalingam et al. introduced a protocol for the large-scale *in vitro* production of iPSC-derived erythroblasts. They generated 0.85 billion cells in a 500 mL spinner flask with cell densities approaching 1.7 × 10^7 cells/mL (Sivalingam et al., 2021). Despite the numerous methods available for producing RBCs from hiPSCs, there is a need for better tools to develop more mature RBCs suitable for human applications and transfusion.

### 4.2 The challenges of hiPSCs-derived red blood cells

Although there have been remarkable advances in the generation of protocols for producing RBCs from human iPSCs, there are some challenges that should be addressed. One of the major challenges is achieving stable expression of β-globin, which is essential for the production of adult hemoglobin (HbA, α2β2) (Wood and Weatherall, 1973). Erythroid cells derived from hiPSCs often express β-globin at low levels. Therefore, to improve the protocol, the mechanisms for controlling the expression of β-globin and γ-globin in the human body have been explored. It has been reported that during fetal development, HbF (α2γ2) is the main hemoglobin that receives oxygen from the mother’s placenta. However, after birth, there is a switch from γ-globin to β-globin, which explains the predominantly high expression of HbA in adult erythroid cells. This switch is regulated by several key transcription factors. MYB, along with SOX6 and GATA1, have important roles in the activation of KLF1. Activated KLF1, in turn, activates BCL11A, thereby silencing γ-globin expression and promoting β-globin expression (Song et al., 2007; Xu et al., 2010; Ali et al., 2021). Thus, BCL11A is known as the crucial activator of HbF silencing (Frangoul et al., 2021; Jawaid et al., 2010; N. Liu et al., 2018).

Enucleation, the removal of the nucleus, is a critical process during the terminal maturation of RBCs. It is controlled by factors such as cell cycle arrest, chromatin condensation, and nuclear polarization. However, in humans, the molecular mechanism underlying the process of enucleation is still not fully understood. Although protocols for generating RBCs from hiPSCs have revealed that enucleation occurs, the enucleation rate needs to be improved. In a study by Dorn et al., in 2015, the enucleation rate of hiPSC-derived RBCs was reported to be around 21%–29% (Dorn et al., 2015). In a recent study, Olivier and his team presented an enhanced protocol that showed notable progress in the expansion and enucleation rates (42%) for erythroid cells. This was accomplished by utilizing a chemically defined culture system without albumin and with reduced transferrin supplementation (Olivier et al., 2019). Additionally, they implemented a purification step to eliminate extruded nuclei and nucleated erythroblasts, leading to a final enucleation rate of 94% (Olivier et al., 2019). Nonetheless, for clinical applications, ensuring safety and achieving large-scale production of hiPSC-derived RBCs remain significant concerns. The implementation of GMP is crucial as it will ensure safe and reproducible processes with minimal variability between batches. Under research settings, hiPSC-derived RBCs are often cultured with BSA or feeder cells, which can cause instability during large-scale production (Lim et al., 2021). Therefore, protocols that utilize mammalian cell culture in bioreactors, which is commonly used in the biopharmaceutical industry, should be adopted to provide more stable and scalable production methods. To establish hiPSC-derived RBCs as a feasible therapeutic product, several significant challenges must be effectively addressed, including ensuring safety, achieving reproducibility, and developing cost-effective protocols for ultra-high density cell culture, efficient differentiation, and maturation. Although much progress has been made, further research and optimization are necessary before hiPSC-derived RBCs can be widely used in clinical applications (Larochelle, 2013; Focosi and Amabile, 2017; Seo, Shin, Kim and Kim, 2019).

## 5 Discussion

The potential of hiPSC-derived blood cells in healthcare, including cell therapy and blood transfusion substitutes, is incredibly promising. Several culture systems have been devised to produce functional RBCs and immune cells from hiPSCs, ranging from using feeder cells to forming EBs, and adopting feeder-free and xeno-free systems. Nevertheless, there are hurdles to overcome in the clinical implementation of hiPSC-derived blood and immune cells. One major challenge is the lack of strategy to increase the efficiency of differentiation protocols. Many protocols are still unable to produce an adequate number of functional RBCs, primarily due to unstable β-globin expression and enucleation. HiPSC-derived immune cells are also limited by the issue of differentiation efficiency. Moreover, the differentiation efficiency of iPSCs may vary depending on their origin or cell type. iPSCs derived from different somatic cell sources (e.g., skin cells, blood cells) may exhibit variations in their differentiation capacity towards specific lineages. Moreover, the choice of the starting material or cell type for generating hiPSCs can affect the subsequent differentiation outcomes (Goldenson et al., 2020). Selecting appropriate starting materials for hiPSC-derived cell production is therefore essential to ensuring the quality and quantity of the final products.

Second, safety and large-scale production are also significant concerns in the clinical applications of iPSCs-derived therapies. The tumorigenicity and toxicity of iPSCs pose a safety risk (Yamanaka, 2020). Additionally, genetic modifications, such as CRISPR/Cas9, used in the production of CAR iPSC-derived T/NK/iNK cells, raise questions about product safety (Chen et al., 2019; Aquino-Jarquin, 2021). To guarantee the safety of hiPSC-derived blood cells, it is imperative to employ standardized techniques for molecular characterization and functional assessment, ensuring the accurate identification, purity, safety, and effectiveness of the generated cells. Adhering rigorously to GMP standards is essential for establishing robust and consistent processes across different batches. While the utilization of bioreactors facilitates large-scale *in vitro* cell culture, it also introduces challenges due to the intricate steps involved, the variety of factors required, and the demands of industrial-scale production. These challenges necessitate the search for recognized standards and dependable quality control systems.

Finally, cost-effectiveness is another important consideration. The use of multiple cytokines, growth factors, and lengthy-time requirements can hinder the industrial application of iPSC-derived cells. Notably, GMP-grade materials are more expensive than research-grade materials, which makes it difficult to reduce costs and improve efficiency in clinical applications.

Compared to chemical drugs, cellular drugs including iPSC-derived cells have unique therapeutic effects. With the rapid development of cellular therapeutics in recent years, more cellular drugs have been approved by the FDA. While cellular drugs that have received marketing approval still encounter certain challenges.

The cost of cellular drugs, particularly gene drugs, is notably high due to factors such as extensive research and development expenses, production costs, and other considerations. This leads to a selling price that surpasses that of chemical drugs. For instance, CAR-T cell therapy drugs can be priced as high as RMB 1.2 million. The high cost of cell drug causes drug payment problems. To resolve this problem, several key factors should be considered. Firstly, it is imperative to establish an equitable pricing mechanism that accurately mirrors both the research and development costs, as well as the therapeutic efficacy of the drugs. Secondly, there should be a concerted effort to broaden the scope of health insurance coverage, thereby enabling more patients to avail themselves of cell therapy drugs and alleviating their financial strain. Moreover, public health systems and governments may contemplate extending financial assistance or devising suitable subsidy policies to bolster the advancement and utilization of cell therapy drugs.

Cell therapy drugs, including iPSC-derived cells, are considered a new and promising treatment approach. Due to the personalized and complex preparation process, cell drugs require highly specialized and experienced healthcare professionals to administer and manage. Firstly, healthcare professionals need to have a deep understanding and mastery of fields such as cell biology, genetics, and immunology to comprehend the principles, safety, and efficacy evaluation of cell therapy drugs. Secondly, healthcare professionals require high proficiency in laboratory techniques and skills, including cell culture, quality control, and analysis. They must possess the capacity to accurately execute the preparation procedures, monitor cell status and quality, and promptly address any potential issues that may arise. Furthermore, healthcare professionals need to have good communication and collaboration abilities, as the preparation of cell therapy drugs often involves cooperation among multiple departments and teams. They should be capable of forming strong collaborations with professionals including cell biologists, geneticists, immunologists, and clinical doctors to ensure the effective execution of treatment strategies. However, this can pose challenges for the clinical integration of cell therapy drugs.

Despite the challenges, significant progress has been made in the generation of hiPSC-derived blood cells. The advent of iPSC technology has opened up new opportunities for establishing next-generation medicines. Although many obstacles remain, significant advancements in this field are feasible in the future.
